# Role of GABRD Gene Methylation in the Nucleus Accumbens in Heroin-Seeking Behavior in Rats

**DOI:** 10.3389/fphar.2020.612200

**Published:** 2021-01-21

**Authors:** Qingxiao Hong, Wenjin Xu, Zi Lin, Jing Liu, Weisheng Chen, Huaqiang Zhu, Miaojun Lai, Dingding Zhuang, Zemin Xu, Dan Fu, Wenhua Zhou, Huifen Liu

**Affiliations:** ^1^ Laboratory of Behavioral Neuroscience, Ningbo Kangning Hospital, Ningbo Institute of Microcirculation and Henbane, School of Medicine, Ningbo University, Ningbo, China; ^2^ Key Laboratory of Addiction Research of Zhejiang Province, Ningbo, China

**Keywords:** heroin, nucleus accumbens, GABRD, methylation, reinstatement

## Abstract

Epigenetic modifications such as DNA methylation play important roles in regulating gene expression and may mediate neuroplasticity and lead to drug-induced aberrant behaviors. Although several brain regions and neurobiological mechanisms have been suggested to be involved in these processes, there is remarkably little known about the effects of DNA methylation on heroin-seeking behavior. Using a Sprague-Dawley rat model, we show that heroin self-administration resulted in gamma-aminobutyric acid type A receptor subunit delta (GABRD) gene hypomethylation, which was associated with transcriptional upregulation of GABRD in the nucleus accumbens (NAc). Systemic l-methionine (MET) administration significantly strengthened the reinstatement of heroin-seeking behavior induced by heroin priming, whereas intra-NAc injections of the DNA methyltransferase (DNMT) inhibitor 5-aza-2′-deoxycytidine (5-Aza-dC) had the opposite effect on heroin-seeking. Meanwhile, 5-Aza-dC treatment decreased DNA methylation and upregulated the expression of GABRD in the NAc, whereas MET had the opposite effect. Our results also reveal that 5-Aza-dC might alter the methylation landscape of the GABRD gene by directly repressing DNMT1 and DNMT3A expression. Furthermore, reinstatement of heroin-seeking behavior was significantly inhibited by directly overexpressing GABRD and remarkably reinforced by GABRD gene silencing in the NAc. Collectively, these results suggest that targeting the GABRD gene and its methylation might represent a novel pharmacological strategy for treating heroin addiction and relapse.

## Introduction

Heroin addiction is a chronic and relapsing brain disorder characterized by compulsive drug-seeking and the negative emotional state of withdrawal. Relapse to drug-seeking after abstinence has long been a challenge in the treatment of heroin addiction. Heroin addiction is characterized by specific behavioral alterations, indicating long-lasting alterations in gene and protein expression within specific reward-related brain regions. Accumulating evidence has demonstrated that epigenetic mechanisms such as DNA methylation regulate drug-induced gene expression profiles and enduring behavioral phenotypes (Nestler, 2014; Kalda and Zharkovsky, 2015). Recent data suggest that altered DNA methylation may indicate the changed gene expression programs in response to experience (Meaney and Szyf, 2005; Klengel et al., 2013) and regulate the synaptic plasticity as well as be implicated in memory formation (LaPlant et al., 2010; Zovkic et al., 2013). Epigenetic modifications may also be a critical molecular mechanism that indicated the enduring addiction-related changes in brain plasticity (Robison and Nestler, 2011; Godino et al., 2015; Massart et al., 2015).

DNA methyltransferases (DNMTs) are responsible for adding methyl groups to cytosine-guanine dinucleotides (CpGs) in the genome (Suzuki and Bird, 2008). In mammals, the main DNMTs include DNMT1 which is responsible for DNA methylation maintenance and another two “*de novo*” methyltransferases that establish new methylation patterns (DNMT3A and DNMT3B) (Ribas et al., 2017). CpG islands methylation recruits co-repressor complexes to interfere with the binding of transcription factor to target DNA sequences (Jaenisch and Bird, 2003). Considerable available evidence demonstrates that drug-induced changes in gene expression are modulated by DNA methylation. Tian et al. (Tian et al., 2012) found that rewarding effects induced by cocaine could be significantly attenuated by reversing the global DNA hypomethylation. Besides, the hippocampus of juvenile rats exposed *in utero* to cocaine shows altered patterns of global DNA methylation and changed gene transcription, for example, genes encoding G-protein coupled receptor 73 (GPR73), polo-like kinase 2 (PLK2) and protein-tyrosine phosphatase non-receptor type 5 (PTPN5) (Novikova et al., 2008). Changes in gene expression occurs after cocaine self-administration, and the changes is shown to be correlate with increased expression of the methyl CpG-binding protein MeCP2 (Host et al., 2011). Additionally, methamphetamine administration may alter DNA methylation patterns through regulation of DNMT1 mRNA levels (Numachi et al., 2007). Interestingly, a recent clinical study showed that methamphetamine-induced changes in long interspersed element-1 methylation are associated with methamphetamine-induced paranoia and therefore may partially explain the pathophysiology of this type of psychosis (Kalayasiri et al., 2019). Previous preclinical study reported that κ1 opioid receptor (OPRK1) promoter methylation was significantly correlated with the length and frequency of drug use in Chinese male heroin addicts (Ji et al., 2018). Xu et al. (2016) demonstrated that methylation level of one CpG sites in brain derived neurotrophic factor (BDNF) promoter was significantly associated with addictive phenotypes of heroin-dependent individuals, including tension-anxiety, anger-hostility, fatigue-inertia, and depression-dejection. However, there are relatively few reports on animal model relevant to heroin addiction, little is known about the role of DNA methylation regulatory events in mediating the lasting effects of heroin-seeking behavior.

γ-aminobutyric acid (GABA) A receptors (GABAARs) are the crucial inhibitory neurotransmitter receptors in the central nervous system. As a class of transmembrane ligand-gated chloride channels, GABAARs consist of five subunits from the 19 known different subunit isoforms, α1–6, β1–3, γ1–3, δ, ε, θ, π, and ρ1–3 (Whiting, 2003; Olsen and Sieghart, 2009). The channels are usually formed by two α subunits, two β subunits, and one other subunit, and the resulting compositional differences are fundamentally responsible for the different functional and pharmacological properties of the channels (Brickley and Mody, 2012). Given that GABAARs are considered to be key targets for alcohol, sleep-promoting drugs and anaesthetics, their importance in regulating schizophrenia, epilepsy, and drug addiction has been highlighted. Previous studies reported that the expression of some GABAAR subunits, such as GABA type A receptor subunit (GABR) beta2 and GABR alpha2, was regulated by DNA methylation in mood disorders (Wang et al., 2016; Gatta et al., 2017; Ye et al., 2019). Besides, recent data suggest that DNA methylation is functionally correlated with the persistent cocaine-craving and may be partly negatively correlated with gene expression changes, for example, that of the GABR delta (GABRD) gene. Furthermore, a previous study showed that mRNA and protein levels of the GABRD were negatively modulated by promoter methylation in the cerebellum of individuals with alcohol use disorder (Gatta et al., 2017). However, no study has reported a correlation between GABRD methylation and heroin addiction.

In the present study, we combine global whole-genome and candidate gene approaches to investigate the effect of DNA methylation on heroin-seeking behavior by analysing DNA methylation profiles and the correlated gene expression changes in the NAc of rats. Our research also explores whether DNA methylation regulates GABRD gene transcription in the NAc after repeated heroin treatment, and whether the pharmacological enhancement or inhibition of DNMTs alters heroin self-administration and the reinstatement of heroin-seeking behavior in rats. Finally, we investigate whether overexpression or down-regulation of GABRD in the NAc of rats significantly alters the associated cues-induced or heroin priming reinstatement after extinction of heroin self-administration.

## Results

### Global DNA Methylation

**FIGURE 1:**
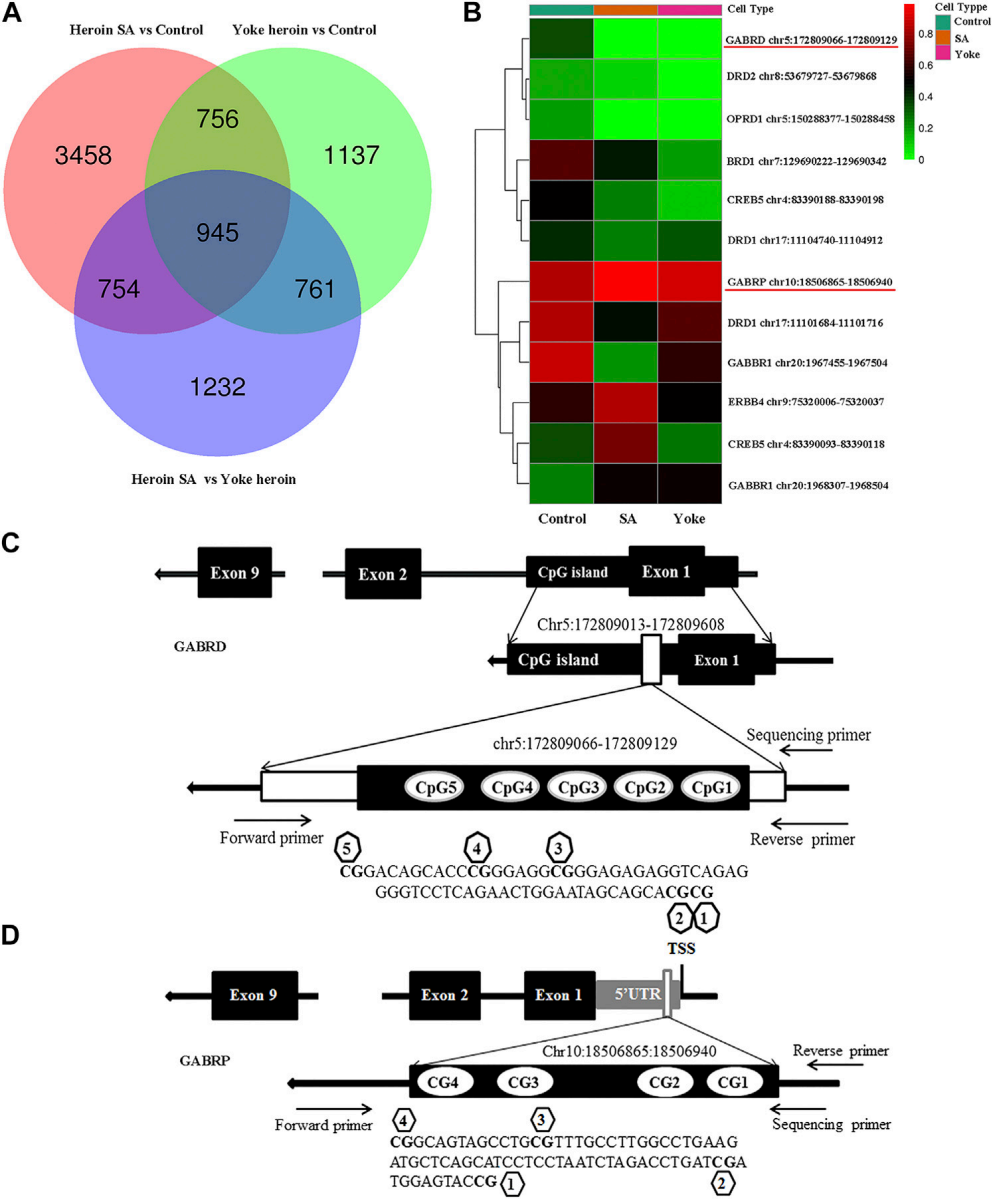
DNA methylation of heroin self-administration rats and the locations of target sequences. **(A)** Venn diagram of promoter-DMRs related genes in the paired comparison of the three groups of rats (*n* = 3 per group). SA: self-administration. **(B)** A hierarchical clustering map of NAc gene methylation for yoked-saline (control), heroin self-administration, and yoked-heroin rats (red, high methylation; green, low methylation). **(C)** The target sequence of GABRD is located in a CpG island which contains 5 CpG sites. **(D)** The target sequence of GABRP is located in the 5′UTR which contains four CpG sites. SA: Self-administration, Control: yoked-saline group.

We performed whole-genome methylation sequencing to investigate changes in methylation profiles after heroin self-administration (PRJNA673675, SRA records will be accessible with the following link after the indicated release date 2024–10–01, https://www.ncbi.nlm.nih.gov/sra/PRJNA673675). On average, more than 331,106,942 sequence reads were obtained per sample, the differentially methylated regions (DMRs) were identified between the three groups. The methylKit and eDMR R package was used for comprehensive DMRs analysis, the eDMR can optimize regional methylation analysis based on bimodal normal distribution and weighted cost function. A comparative analysis revealed that there were 38,177 DMRs between the heroin self-administration and the yoked-saline group, and 23,129 DMRs existed between the yoked-heroin and the yoked-saline group. As shown in Figure 1A, the pink [Heroin Self-administration vs. Yoked-saline group (Control)], green (Yoked-heroin vs. Yoked-saline group) and blue (Heroin Self-administration vs. Yoked-heroin group) areas represent the genes that differentially methylated in promoter regions in the two corresponding groups respectively. And their area of overlap represents the core. In total, the co-existing 1,701 (756 + 945) genes were significantly differentially methylated in their promoter regions in the yoked-saline group compared against the heroin self-administration and the yoked-heroin group, respectively. The core value 945 in the middle represent the DMRs co-existing in the three sets. Some focused hypermethylated and hypomethylated genes are shown in Figure 1B. Of these, the methylation level of the GABRD gene was significantly reduced in the heroin self-administration and yoked-heroin groups compared with that in the yoked-saline group (DMR: chr5:172809066–172809129, located within CpG islands 1 kb downstream of the transcription start site (TSS) (Figure 1C) (*p* < 0.05, *p* < 0.05). Besides, methylation level of the GABRP gene (DMR: chr10:18506865–18506940, located within the 5′UTR) was shown to be increased in the heroin-self-administration compared with that in the yoked-saline group (*p* < 0.05) (Figure 1D).

### Confirmation of the Changes in GABRD and GABRP Gene Methylation in Heroin-Administration Rats

**FIGURE 2:**
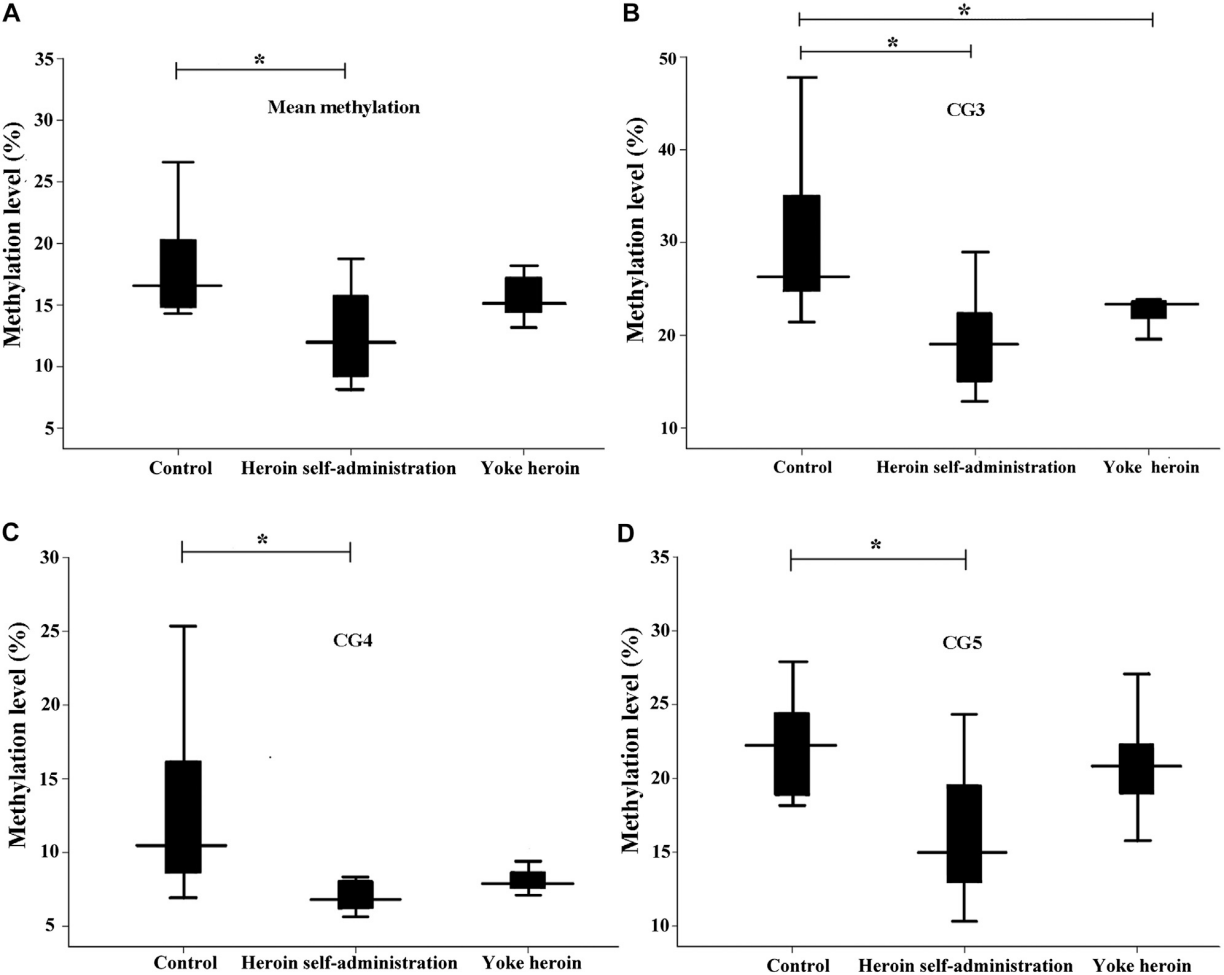
Comparisons of GABRD methylation levels among three groups (*n* = 10 per group); Heroin self-administration group, which received heroin self-administration for 14 days, and iii) yoked-heroin group, which received amounts of heroin equal to those received self-administration over the same course of time (Yoke heroin). The methylation levels were calculated and the statistical differences evaluated by one-way ANOVA followed by Student–Newman–Keuls post hoc comparisons. Data were presented as means ± SDs. *p < 0.05.

To further confirm the results, pyrosequencing was performed. Significant correlations among five CpG sites in GABRD were observed (*r* > 0.50, *p* < 0.05). Therefore, the average percentage value for 1–5 CpG sites were used for subsequent analysis. The average GABRD methylation levels were significantly reduced in heroin self-administration group as compared to those in the yoked-saline group [*F*_(2,26)_ = 5.91, *p* = 0.008; Figure 2A]. Further analysis of different CpG cites showed that methylation levels at CG3, CG4, and CG5 sites were decreased in the heroin self-administration group compared with those in the yoked-saline group [CpG3: *F*_(2,26)_ = 7.82, *p* = 0.002, CpG4: *F*_(2,26)_ = 4.95, *p* = 0.015, CpG5: *F*_(2,26)_ = 6.18, *p* = 0.006, Figures 2B–D]. Additionally, the CG3 site exhibited reduced methylation in yoked-heroin group compared with that in the yoked-saline group (*p* = 0.042). However, the CG1 and CG2 sites exhibited no changes [CpG1: *F*_(2,26)_ = 2.71, *p* = 0.085, CpG2: *F*_(2,26)_ = 5.91, *p* = 0.058].

**FIGURE 3:**
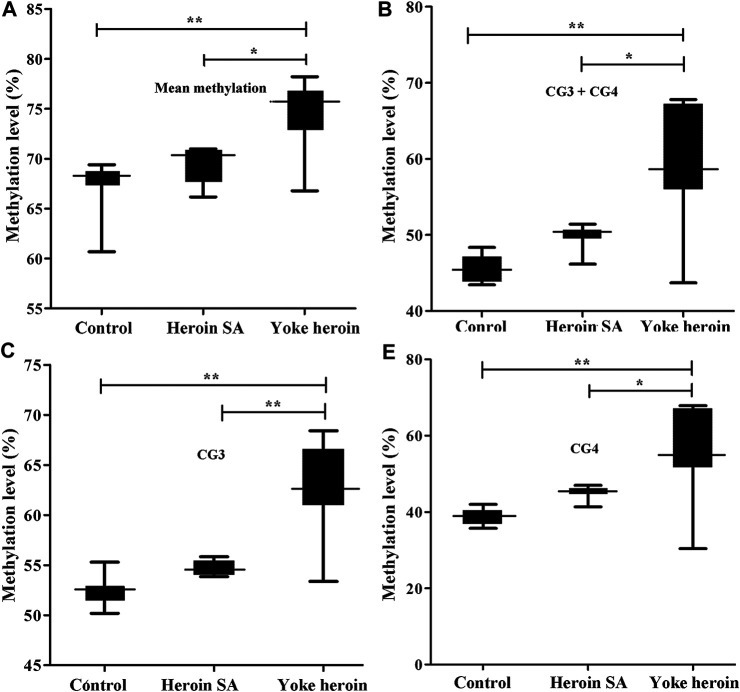
Comparisons of GABRP methylation levels among the three groups (*n* = 10 per group). SA: Self-administration. The methylation levels were calculated and the statistical differences evaluated by one-way ANOVA followed by Student–Newman–Keuls post hoc comparisons. Data were presented as means ± SDs. *p < 0.05; **p < 0.001.

The sequence of the GABRP promoter that contains four CpG sites is shown in Figure 1D. Significant correlations between CG1 and CG2 were observed (*r* = 0.63, *p* = 1.08E−4); besides, there were significant correlation between CG3 and CG4 (r = 0.92, *p* = 1.62E−13). In total, the average methylation levels of GABRP were obviously increased in heroin self-administration and yoked-heroin groups compared with that in the yoked-saline group [*F*_(2,26)_ = 16.77, *p* = 1.45E−5, Figure 3A]. Meanwhile, the methylation level at the CG3 site was significantly increased in yoked-heroin group compared with those in yoked-saline group and heroin self-administration groups [F_(2,29)_ = 38.80, *p* = 6.34E−9, Figure 3C]. Moreover, there were statistically significant differences in CG3 + CG4 sites and CG4 sites among the three groups [F_(2,29)_ = 23.41, *p* = 8.87E−7, Figure 3B; *F*_(2,29)_ = 16.62, *p* = 1.55E−5, Figure 3D]. Significant GABRP hypermethylation in heroin-self-administration and yoked-heroin groups, compared with that in the yoked-saline group were detected.

**FIGURE 4:**
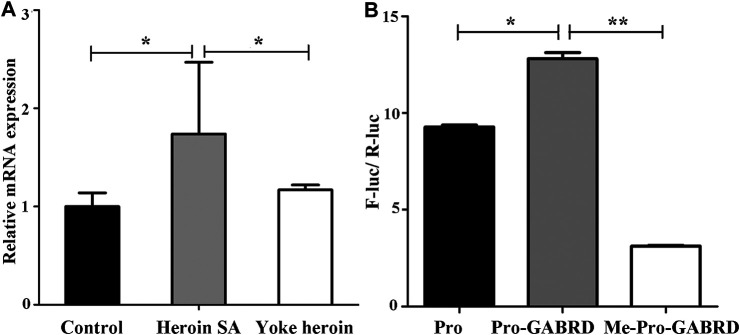
GABRD mRNA expression level and enhancer-like activity of target sequence. **(A)** Relative mRNA expression of GABRD in the three groups (Control, *n* = 6; Heroin SA, *n* = 6; Yoke heroin, *n* = 5). **(B)** Dual luciferase reporter gene assay for GABRD (*n* = 3 per group). Control: Yoked-saline group; SA: Self-administration; Pro: pGL3-promoter vector; Me: Methylation; F-Luc: Firefly luciferase; R-Luc: Renilla luciferase. The relative mRNA expression of GABRD and the fluorescence ratios among three groups were compared using one-way ANOVA followed by Student–Newman–Keuls post hoc comparisons. The Data were presented as means ± SDs. *p < 0.05; **p < 0.001.

The relative mRNA expression of GABRD and GABRP were determined by RT-qPCR; one-way ANOVA showed that GABRD expression was significantly different in the three groups [*F*_(2,14)_ = 4.86, *p* = 0.025]. The multiple comparisons illustrated that GABRD mRNA expression in the heroin self-administration group increased compared with that in the yoked-saline and yoked-heroin groups (*p* = 0.031 and *p* = 0.012, respectively Figure 4A). Whereas GABRP mRNA expression remained unchanged in three groups [*F*_(2,14)_ = 0.056, *p* = 0.946]. These results revealed a significant negative correlation between GABRD gene methylation and mRNA expression in the NAc.

### Detection of Enhancer Activities of the GABRD Fragments

Results of the double luciferase reporter gene assay showed that the fluorescence ratios among the pGL3-promoter, pGL3-promoter-GABRD, and Me-pGL3-promoter-GABRD groups were significantly different [*F*_(2,6)_ = 1.97E3, *p* = 3.51E−9]. The post hoc comparisons showed fluorescence ratios in pGL3-promoter-GABRD which contains the GABRD segment that included five CpG sites were significantly higher than that in basal reporter plasmids (pGL3-promoter vector). These results revealed that the candidate fragment of GABRD had somehow enhancer-like activity (*p* < 0.05, Figure 4B). We then investigated the fluorescence ratios in the unmethylated (Pro-GABRD) or methylated forms of GABRD segment (Me-Pro-GABRD), the result demonstrated that fluorescence activity was significantly reduced in Me-Pro-GABRD compared with that in Pro-GABRD, these findings provided evidence that the methylated form of GABRD can weaken the gene activity significantly (*p* < 0.001, Figure 4B).

### The Effects of MET on the Acquisition of Heroin Self-Administration and Heroin Reinstatement

**FIGURE 5:**
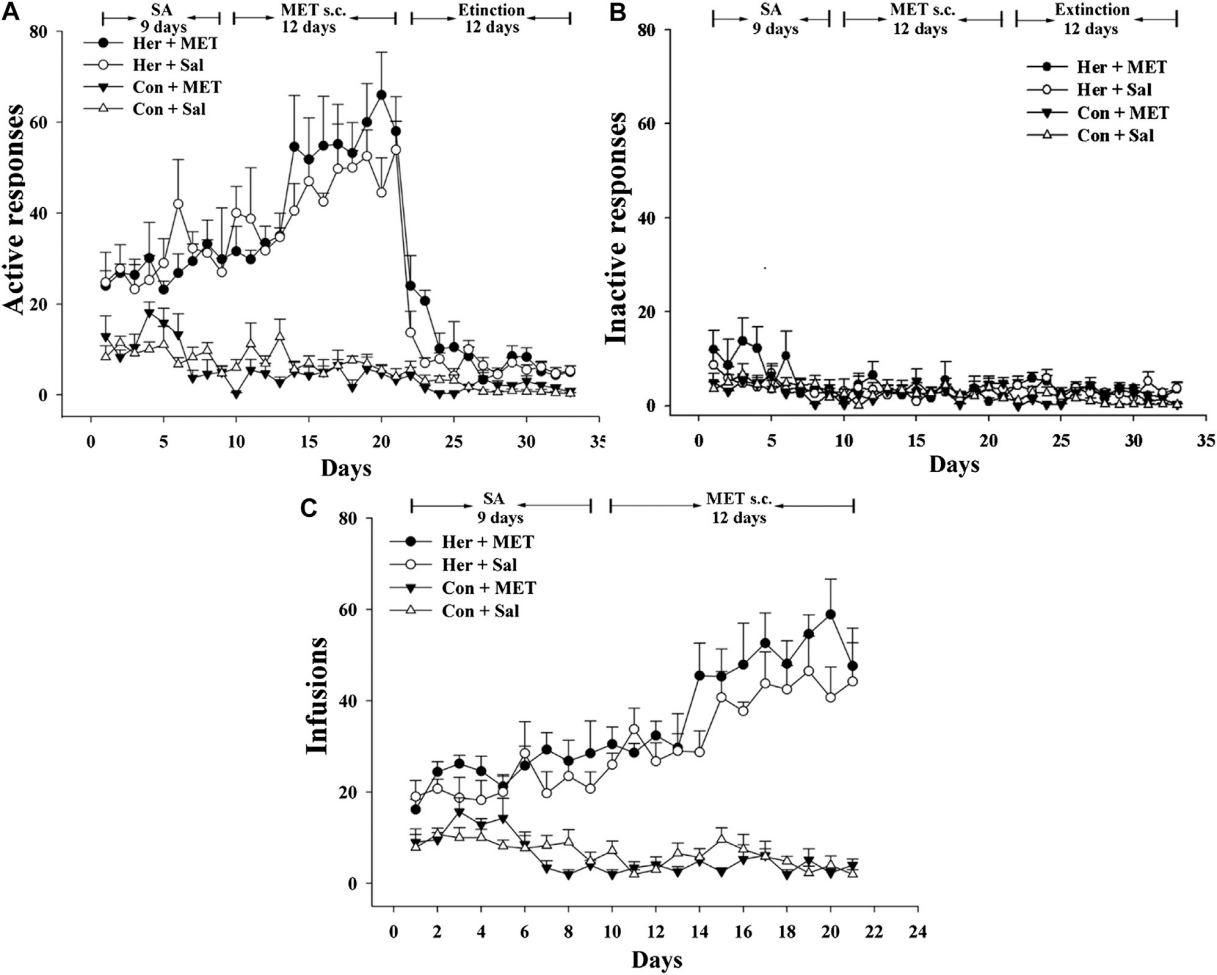
Effect of systemic injection of MET on heroin self-administration (*n* = 5 per group). **(A)** Active, and **(B)** inactive nose-poke responses during training. **(C)** The number of infusions per session. Her + MET: heroin self-administration and MET-treated rats, Her + Sal: heroin self-administration and saline-treated rats, Con + MET: saline self-administration and MET-treated rats, Con + Sal: saline self-administration and saline-treated rats. The active, inactive nose-poke responses and infusions were measured by two-way repeated-measures ANOVA with treatment group and time as factors. Data were expressed as means ± SDs. SA: self-administration; s.c.: subcutaneous injection; MET: l-methionine; Sal: Saline.

**FIGURE 6:**
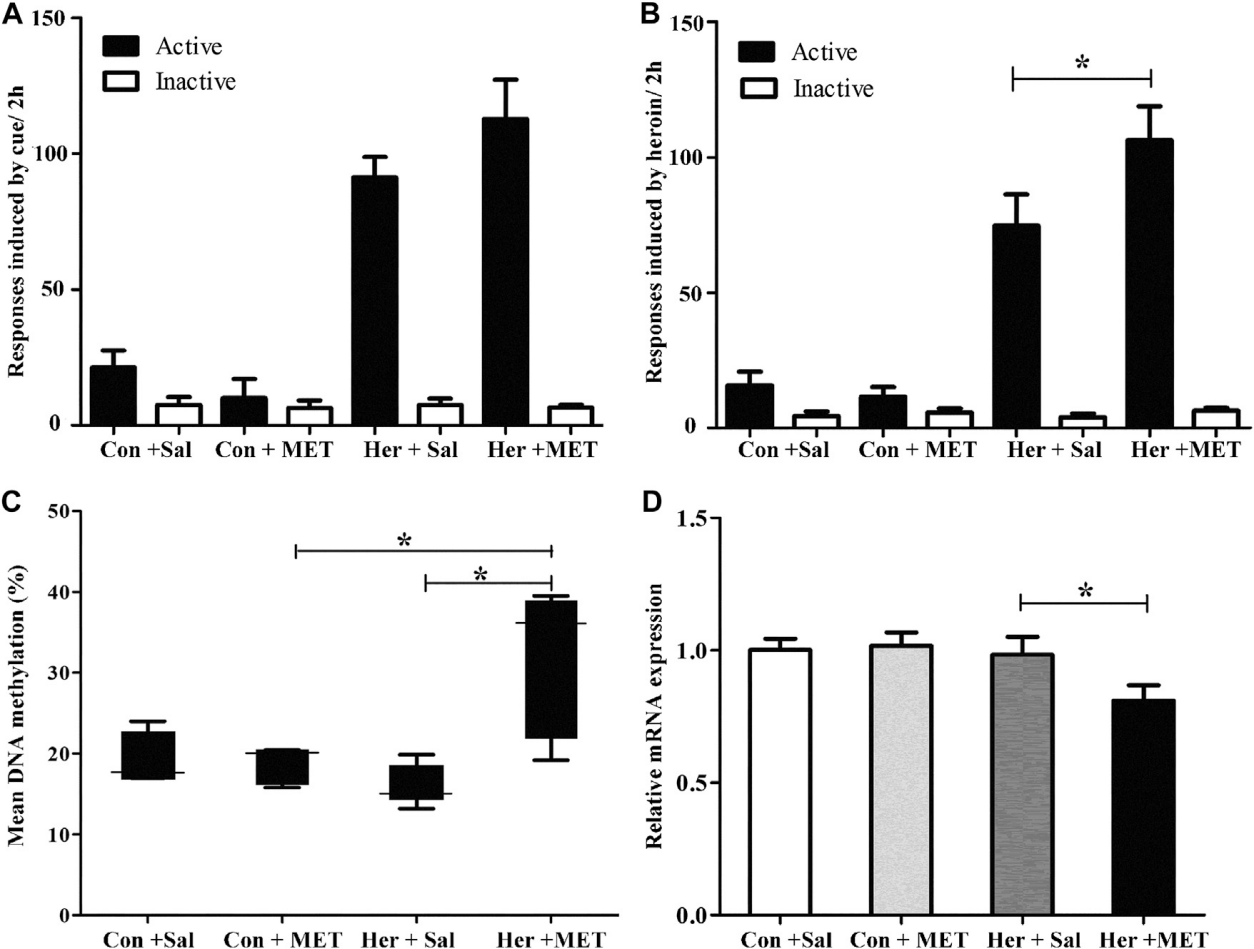
Effect of systemic injection of MET on the reinstatement of heroin-seeking behavior and GABRD gene methylation and mRNA expression levels. **(A)** Systemic injection of MET had no effect on reinstatement of heroin-seeking behavior induced by heroin priming. **(B)** Systemic injection of MET enhanced reinstatement of heroin-seeking behavior induced by cues. **(C)** Systemic injection of MET increased the gene methylation of GABRD in heroin self-administration group. **(D)** Systemic injection of MET decreased the mRNA expression level of GABRD in heroin self-administration group. Con: saline self-administration; Her: Heroin; MET: l-methionine; Sal: Saline. The active and inactive nose-poke responses were measured by two-way repeated-measures ANOVA with treatment group and time as factors. Two-way ANOVA was used to analyze methylation level or mRNA expression of GABRD, with MET treatment and heroin self-administration as the independent variables. Data were expressed as means ± SDs. *p < 0.05.

Rats was injected with MET (s.c.) at 0.5 h prior to heroin self-administration training from the 10th day of training for 12 consecutive days. The two-way repeated-measures ANOVA indicated that the number of infusions and active nose-poke responses during heroin self-administration training after MET treatment showed no significant statistical differences [*F*_(1,9)_ = 4.175, *p* = 0.071] [*F*_(1,9)_ = 2.176, *p* = 0.157, Figures 5A–C]. Notably, MET-treated rats had significantly higher number of active nose-poke responses during the reinstatement induced by heroin priming compared with saline-treated rats [*F*_(1,27)_ = 4.890, *p* = 0.036, Figure 6B] and showed an increasing trend in number of active nose-poke responses during the reinstatement induced by heroin cues; however, these differences were not statistically significant [*F*_(1,29)_ = 0.507, *p* = 0.485, Figure 6A].

### The Effects of MET on Regulation of GABRD Gene Methylation and mRNA Expression

Pyrosequencing was used to determine the effects of methyl supplementation on GABRD methylation. Figure 6C showed the methylation state of the five GABRD CpGs in the NAc. Significant correlations among these five CpG sites were observed (r > 0.87, *p* < 0.001). Two-way ANOVA revealed a significant main effect for MET [*F*_(1, 17)_ = 5.41, *p* = 0.036] and interaction (MET and heroin) [*F*_(1, 17)_ = 6.54, *p* = 0.023] on GABRD gene methylation; however, no effect of heroin on methylation was observed [*F*_(1, 17)_ = 0.574, *p* = 0.461]. Thereafter, post hoc multiple comparisons were performed, and the results showed that the GABRD gene exhibited an overall increase in DNA methylation in the Her + MET group, and there were significant differences between Her + MET vs. Her + Sal (*p* = 0.003), Her + MET vs. Con + MET (*p* = 0.034), and Her + MET vs. Con + Sal (*p* = 0.047) groups (Figure 6C). Additionally, there were significant effects of MET at CpG2 [*F*_(1, 17)_ = 4.87, *p* = 0.045], CpG3 [*F*_(1, 17)_ = 5.83, *p* = 0.030], and CpG4 [*F*_(1, 17)_ = 6.54, *p* = 0.023]. Meanwhile, significant interaction of MET treatment and heroin effects at CpG3 [*F*_(1, 17)_ = 6.87, *p* = 0.02] and CpG5 [*F*_(1, 17)_ = 6.41, *p* = 0.024] were detected. Conversely, contrary to the methylation levels, mRNA expression level in the Her + MET group was obviously lower than that in the Her + Sal group [*F*_2, 28)_ = 3.686, *p* = 0.038, Figure 6D].

### The Effects of 5-Aza-dC on Heroin Priming Reinstatement

**FIGURE 7:**
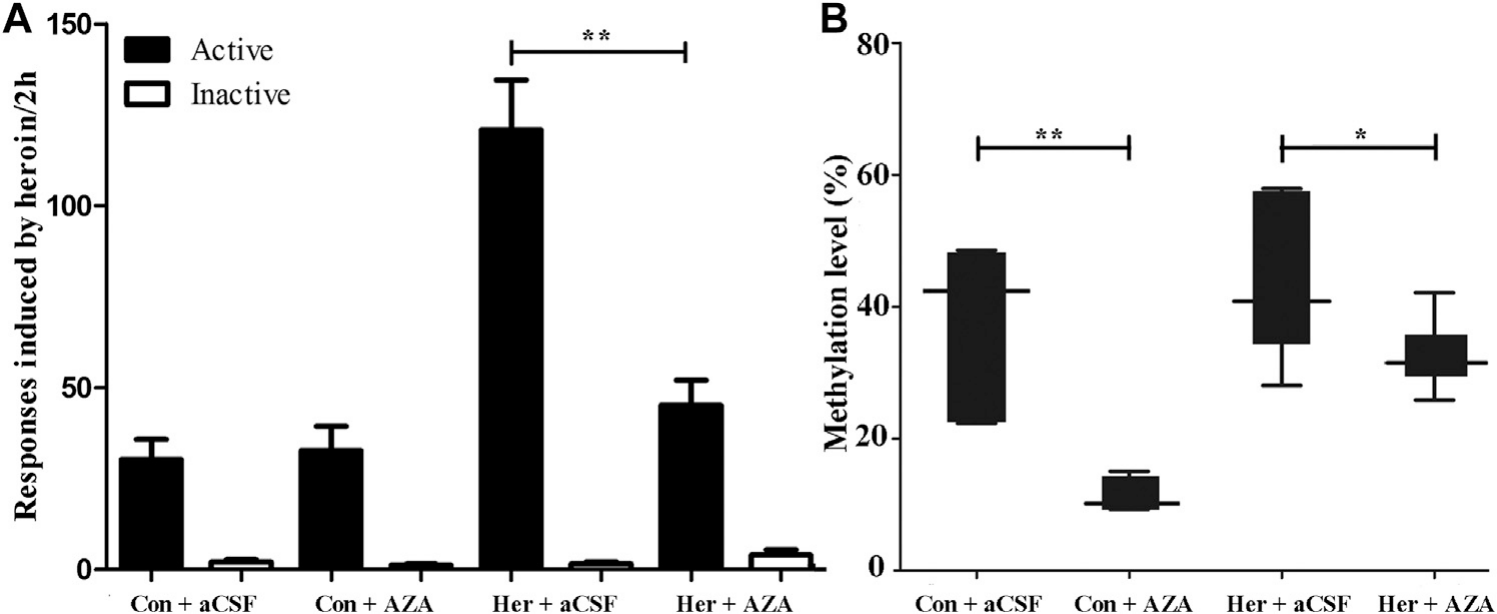
Effect of intra-NAc microinjection of 5-Aza-dC on the reinstatement of heroin-seeking and GABRD gene methylation (*n* = 5 per group). **(A)** Intra-NAc microinjection of 5-Aza-dC abolished reinstatement of heroin-seeking behavior induced by heroin priming. **(B)** Intra-NAc microinjection of 5-Aza-dC inhibited the DNA methylation of GABRD. Con: saline self-administration; Her: Heroin; aCSF: artificial cerebrospinal fluid solution; AZA: 5-Aza-dC, 5-Aza-2-deoxycytidine. The active and inactive nose-poke responses were measured by two-way repeated-measures ANOVA with treatment group and time as factors. Two-way ANOVA followed by Student–Newman–Keuls post hoc comparisons was used to analyze methylation level of GABRD, with AZA treatment and heroin self-administration as the independent variables. Data were expressed as means ± SDs. *p < 0.05; **p < 0.001.

Given the ability of MET to reinforce the reinstatement of heroin-seeking induced by heroin priming, we hypothesized that microinjection with the methyltransferase inhibitor 5-Aza-dC might attenuate the reinstatement of heroin-seeking. Thus, we treated rats with intra-NAc microinjection of 5-Aza-dC or aCSF in the last 1, 3, 5 days of extinction training, followed by heroin priming reinstatement testing. The two-way ANOVA analysis revealed significant main effects of 5-Aza-dC [*F*_(1, 19)_ = 14.402, *p* = 0.001], heroin [*F*_(1,19)_ = 28.475, *p* = 5.46E−5], and interaction (heroin and 5-Aza-dC) [*F*_(1,19)_ = 16.367, *p* = 8.40 E−4] on active responses during heroin reinstatement. Furthermore, the post hoc analysis showed that heroin priming reinstatement was prevented by pre-treatment with 5-Aza-dC (Her + AZA vs. Her + aCSF, *p* = 1.47 E−4, Figure 7A). Meanwhile, the active responses of heroin reinstatement obviously increased in the Her + aCSF group compared with that in the Con + aCSF group (*p* = 9.06 E−6) (Figure 7A).

### The Effects of 5-Aza-dC on Methylation and the Expression of GABRD and DNMTs in the NAc

**FIGURE 8:**
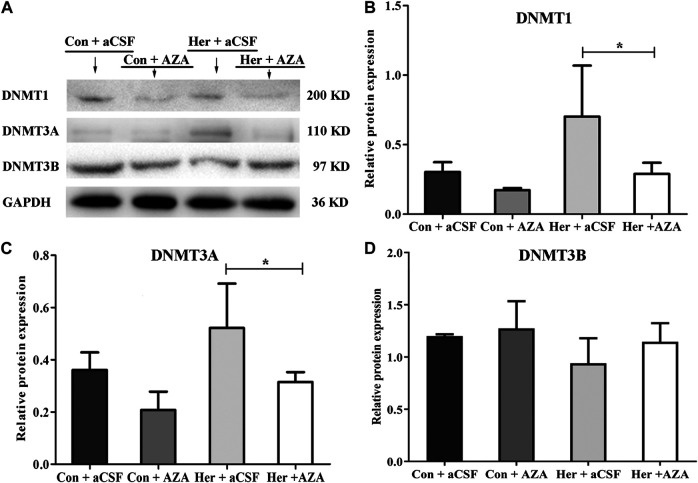
Effect of 5-Aza-dC treatment on DNMT protein expression (*n* = 5 per group). **(A)** Effect of intra-NAc microinjection of 5-Aza-dC on the protein expression levels of DNMTs. **(B)** Intra-NAc microinjection of 5-Aza-dC abolished DNMT1 expression in the heroin-self-administration group. **(C)** Intra-NAc microinjection of 5-Aza-dC abolished DNMT3A expression in the heroin-self-administration group. **(D)** Intra-NAc microinjection of 5-Aza-dC had no effect on DNMT3B expression in four groups. Con: saline self-administration; Her: Heroin; aCSF: artificial cerebrospinal fluid solution; AZA: 5-Aza-dC, 5-Aza-2-deoxycytidine. The groups were compared using two-way ANOVA followed by Student–Newman–Keuls post hoc test. Data were expressed as means ± SDs. *p < 0.05.

Pyrosequencing was used to investigate the effect of 5-Aza-dC on DNA methylation. The results revealed significant correlations among the five GABRD CpG sites (*r* > 0.743, *p* < 0.001). Two-way ANOVA showed a significant main effect of 5-Aza-dC [*F*_(1, 21)_ = 21.83, *p* = 1.89E−4] and heroin [*F*_(1, 21)_ = 12.99, *p* = 0.002] on the average methylation levels of these five CpG sites. Meanwhile, there was significant effect of 5-Aza-dC at the CpG1 [*F*_(1, 21)_ = 10.95, *p* = 0.004], CpG2 [*F*_(1, 21)_ = 8.55, *p* = 0.009), CpG3 [*F*_(1, 21)_ = 30.60, *p* = 2.98E−5], CpG4 [*F*_(1, 21)_ = 12.71, *p* = 0.002], and CpG5 [*F*_(1, 21)_ = 33.42, *p* = 1.77E−5] sites. Simultaneously, significant effects of heroin at CpG1 [*F*_(1, 21)_ = 11.64, *p* = 0.003], CpG2 [*F*_(1, 21)_ = 13.97, *p* = 0.002], CpG3 [*F*_(1, 21)_ = 16.59, *p* = 0.001], CpG4 [*F*_(1, 21)_ = 4.64, *p* = 0.045], and CpG5 [*F*_(1, 21)_ = 10.35, *p* = 0.005] were detected. The significant effect of interaction was only indicated at CpG3 [*F*_(1, 21)_ = 10.00, *p* = 0.005]. Furthermore, the multiple comparisons revealed that average methylation levels of the GABRD gene were significantly decreased after treatment with 5-Aza-dC (Her + AZA vs. Her + aCSF, *p* = 0.038, Con + AZA vs. Con + aCSF, *p* = 4.64E−4] (Figure 7B). Additionally, western blotting showed that treatment with 5-Aza-dC significantly altered DNMT1 and DNMT3A expression in NAc, but DNMT3B remained unaffected (Figures 8A–D). Two-way ANOVA revealed that DNMT1 and DNMT3A were down-regulated after 5-Aza-dC treatment in the NAc [*F*_(1,15)_ = 6.10, *p* = 0.029 and *F*_(1,15)_ = 9.79, *p* = 0.009] for main effect of 5-Aza-dC, respectively; *F*_(1,15)_ = 5.50, *p* = 0.037 and *F*_(1,15)_ = 5.40, *p* = 0.038 for main effect of heroin self-administration, respectively. However, no interaction effect 5-Aza-dC and heroin self-administration was observed [*F*_(1,15)_ = 1.65, *p* = 0.223 and *F*_(1,15)_ = 0.218, *p* = 0.649, respectively], but DNMT3B expression remained unaffected [*F*_(1,15)_ = 0.002, *p* = 0.963 for 5-Aza-dC effect; *F*_(1,15)_ = 0.456, *p* = 0.512 for heroin effect; *F*_(1,15)_ = 0.735, *p* = 0.408 for interaction effect]. Post hoc multiple comparisons showed that DNMT1 and DNMT3A levels decreased in the heroin self-administration group after treatment with 5-Aza-dC in the NAc (Her + AZA vs. Her + aCSF, *p* = 0.021, *p* = 0.026, respectively, Figures 8B,C). However, there were decreasing trends of DNMT1 and DNMT3A protein expression levels after pretreatment with 5-Aza-dC in control group, although the differences were not statistically significant (Con + AZA vs. Con + aCSF, *p* = 0.418, *p* = 0.084, respectively, Figures 8B,C). Correspondingly, the level of the GABRD in the NAc was significantly up-regulated after intra-NAc microinjection of 5-Aza-dC (Figurs 9A,B, Heroin + AZA vs. Heroin + aCSF: *p* = 0.018, Con + AZA vs. Con + aCSF: *p* = 0.010).

**FIGURE 9:**
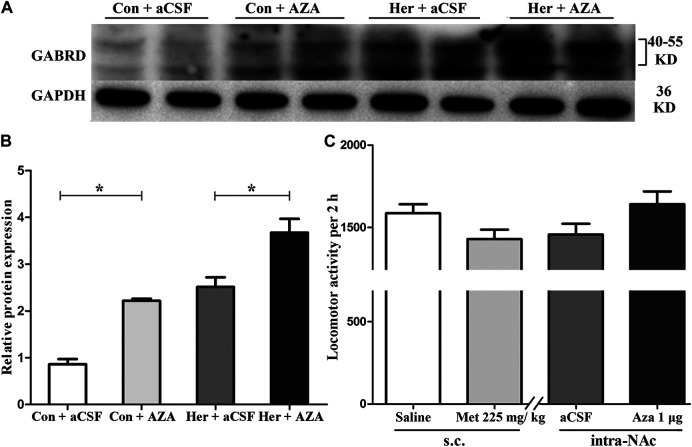
Effect of 5-Aza-dC on the protein expression level of GABRD and effect of MET or 5-Aza-dC treatment on locomotor activity **(A)** and **(B)** Intra-NAc microinjection of 5-Aza-dC increased the protein expression level of GABRD (*n* = 5 per group). **(C)** MET or 5-Aza-dC had no effect on locomotion activity (*n* = 7 per group). Con: saline self-administration; Her: Heroin; aCSF: artificial cerebrospinal fluid solution; AZA: 5-Aza-dC, 5-Aza-2-deoxycytidine; MET: l-methionine; s.c.: subcutaneous injection. The relative protein expression level of GABRD in four groups were compared using two-way ANOVA followed by Student–Newman–Keuls post hoc test. Effect of MET and 5-Aza-dC treatment on locomotion activity were measured by Student t-test. Data were expressed as means ± SDs. *p < 0.05.

### Effect of MET and 5-Aza-dC Treatment on Locomotion Activity

As shown in Figure 9C, statistical analysis revealed no significant effects of either systemic injection of MET or intra-NAc microinjection of 5-Aza-dC on the locomotor activities of rats compared with the corresponding vehicle control [MET vs. Saline_(s.c.)_: 1,428.71 ± 151.36 vs. 1,585.29 ± 147.07, *p* = 0.42; 5-Aza-dC vs. aCSF_(intra-NAc)_: 1,640.86 ± 204.93 vs. 1,456.29 ± 173.56, *p* = 0.28].

### Effect of GABRD Overexpression or RNAi on Heroin Reinstatement

**FIGURE 10:**
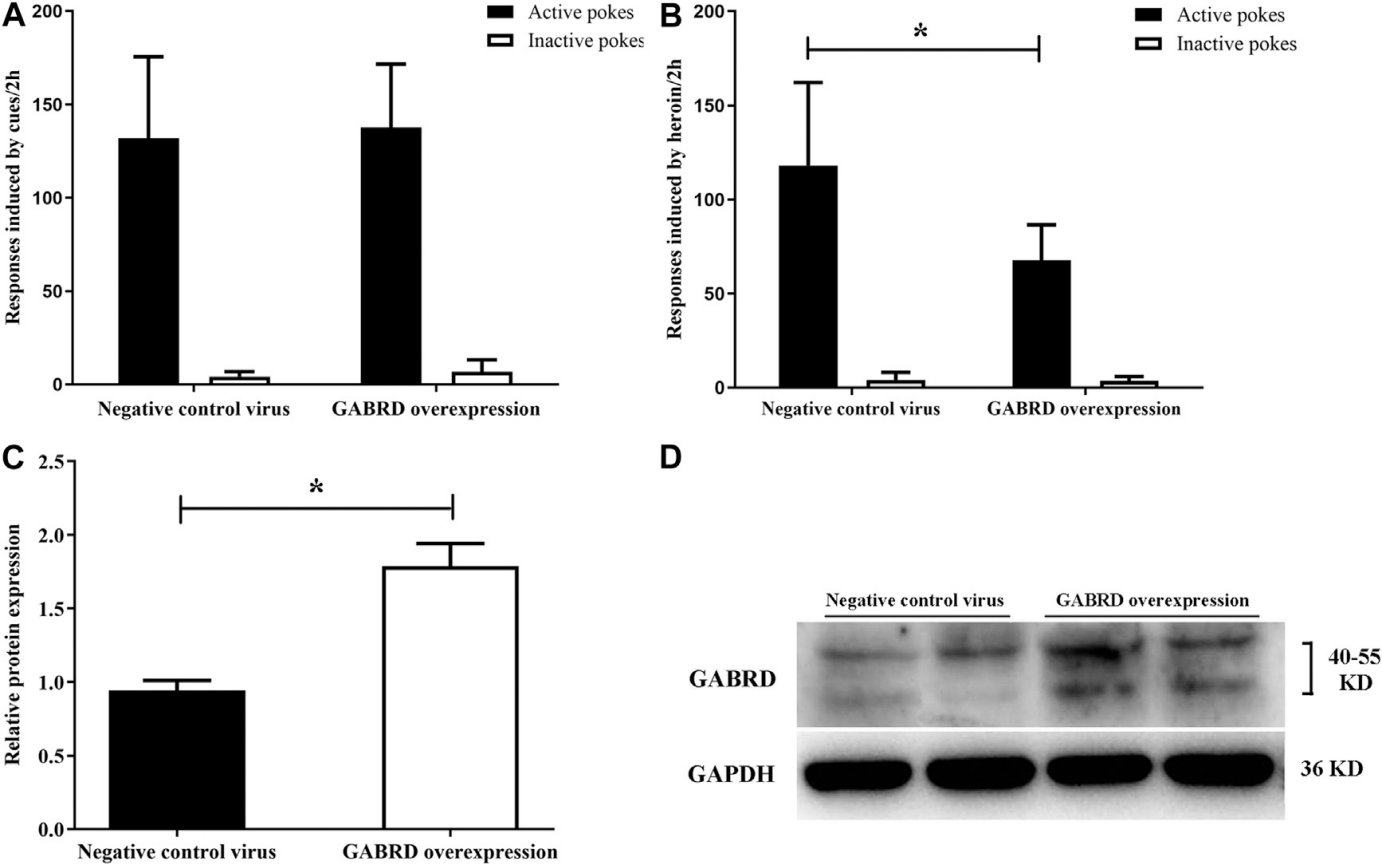
Effects of overexpression of GABRD protein on the reinstatement (*n* = 8 per group). **(A)** Overexpression of GABRD had no effect on cues-induced reinstatement. **(B)** Overexpression of GABRD abolished the heroin priming-induced heroin-seeking behavior. **(C)** Grayscale analysis of protein expression level of GABRD after the overexpression of GABRD. **(D)** Western blot analysis of relative protein expression level of GABRD. Two groups were compared using Student *t*-test. Data were expressed as means ± SDs. *p < 0.05.

We investigated whether GABRD overexpression or RNAi in the NAc of rats would cause changes in heroin reinstatement. Our results showed that over-expression of GABRD in the NAc had no effect on cues-induced reinstatement of heroin-seeking behavior [*F*_(1,14)_ = 0.083, *p* = 0.78, Figure 10A], but the heroin priming reinstatement was significantly inhibited [*F*_(1,14)_ = 8.83, *p* = 0.01, Figure 10B]. At the same time, western blotting showed that the level of GABRD expression in the NAc significantly increased (*p* = 0.038, Figures 10C,D).

**FIGURE 11:**
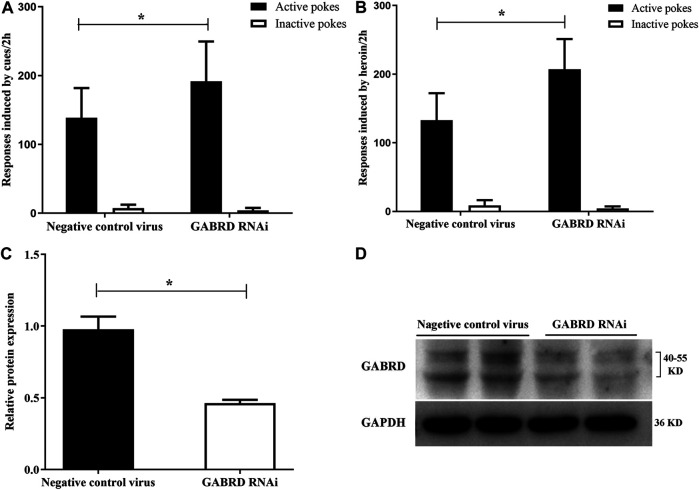
Effects of down-regulation of GABRD on the reinstatement (*n* = 8 per group). **(A)** Down-regulation of GABRD reinforced the cues-induced reinstatement. **(B)** Knockdown of GABRD enhanced the heroin priming-induced heroin-seeking behavior. **(C)** Grayscale analysis of protein expression level of GABRD. **(D)** Western blot analysis of relative protein expression level of GABRD. Two groups were compared using Student *t*-test. Data were expressed as means ± SDs. *p < 0.05.

Our results also suggest that GABRD RNAi in the NAc could significantly enhance the cues-induced or heroin priming reinstatement [*F*_(1,16)_ = 4.86, *p* = 0.04; F_(1,16)_ = 14.33, *p* = 0.002, Figures 11A,B]. Correspondingly, the level of GABRD expression significantly decreased after microinjected with GABRD RNAi AAV vector (*p* = 0.031, Figures 11C,D).

## Discussion

In the present study, we revealed that heroin self-administration induced global DNA methylation changes in the NAc. The DMRs of some GABAARs were identified, which are responsible for mediating the inhibitory effects of GABA. Subsequent validation by pyrosequencing also verified that DNA methylation at the GABRD CpG island significantly decreased and that GABRP promoter methylation significantly increased in the NAc of heroin self-administration rats. Besides, we demonstrated that MET, a methyl donor, had no effect on heroin self-administration, but it could reinforce the reinstatement of heroin-seeking induced by heroin priming while significantly increase DNA methylation levels and decrease mRNA expression of GABRD in the Her + MET group compared with that in the Her + Sal group. Additionally, 5-Aza-dC, a DNA methyltransferase inhibitor, was found to significantly attenuate the reinstatement induced by heroin priming, decrease GABRD methylation levels, and up-regulate its protein expression. With regard to the mechanisms by which the methylation landscape of GABRD was altered by 5-Aza-Dc in the NAc after reinstatement from heroin self-administration, our research found that 5-Aza-dC might alter the methylation landscape of GABRD through directly repressing DNMT1 and DNMT3A expression. Furthermore, 5-Aza-dC and MET had no significant effect on locomotor activity. Since pretreatment with 5-Aza-dC or MET failed to change the locomotor activity in heroin self-administration rats, the heroin induced reinstatement-related changes after intra-NAc injection of 5-Aza-dC or subcutaneous injection with MET might not be caused by impairment of coordinated motor ability or locomotor hypersensitization. Finally, we selectively overexpressed or silenced the GABRD gene in the NAc of rats to detect the effect of GABRD on heroin-seeking behavior. The results demonstrated that over-expression of GABRD in the NAc significantly reduced the reinstatement induced by heroin priming. On the contrary, GABRD RNAi led to an increase in cues-induced or heroin priming reinstatement.

Several genetic and environmental factors are known to contribute to the development of heroin addiction (Randesi et al., 2016). GABA inhibitory neurotransmitter receptors, especially GABAARs, are considered to play a crucial role in multiple drug-elicited neurobehavioral responses and cognitive impairment and to mediate the rewarding effects of drug abuse (Azevedo and Mammis, 2018; Engin et al., 2018; Mitchell et al., 2018). Previous studies have revealed that the NAc GABAergic system is an important mediator of heroin self-administration and that the GABRD receptors primarily control the excitability of the baseline neuronal network through shunting and tonic inhibition (Chuang and Reddy, 2018). Besides, GABRD may be essential for the cognitive and behavioral effects of consumed alcohol, such as behavioral tolerance, anxiolysis, and rewarding and reinforcing effects (Nie et al., 2011). In addition, mutations in GABRD are thought to be related to the pathology of epilepsy (Hernandez and Macdonald, 2019). However, the methylation of GABRD in heroin addiction is rarely mentioned.

In this study, rats were trained to self-administer heroin, and GABRD methylation was found to be significantly decreased after heroin self-administration training. Besides, the DNA methylation level of GABRD increased in the Her + MET group compared with that in the Her + Sal group after systemic injection of MET. On the contrary, intra-NAc microinjection of 5-Aza-dC significantly decreased GABRD gene methylation. Furthermore, gene methylation alterations in GABRD were negatively correlated with the level of GABRD expression; this result may be explained by the fact that the candidate fragment of GABRD has some enhancer-like activity, and consequently the methylated fragment of GABRD led to reduced expression. These data presented here further validate that changes in GABRD expression in the NAc affect the characteristics of heroin addiction in a DNA methylation-dependent manner.

These data were also generally consistent with findings showing that GABRD methylation in the NAc significantly decreased after cocaine self-administration (Massart et al., 2015). Research on cancer had also indicated that methylation in the c.g., 13916816 CpG site of GABRD was negatively correlated with mRNA expression and was related to the overall survival status of adult isocitrate dehydrogenase wild-type diffuse low-grade glioma patients (Zhang et al., 2019). However, there had been few reports regarding the correlation between GABRP methylation and drug addiction. In the present study, our results showed that GABRP methylation was elevated in the NAc of heroin self-administration rats; however, mRNA expression remained unchanged during heroin addiction. Our results were partly consistent with those of some former studies—for example, Zhao et al. (2013) showed that CpG methylation of the GABRP promoter in alcohol dependence (AD) patients was higher than that in their AD-discordant siblings; moreover, this was accompanied by functional GABRP downregulation. In the present study, the most important finding was that the DNA methylation status of the GABRD or GABRP genes, especially the GABRD gene, might serve as biomarkers for evaluating the severity of addiction or addiction-like behavior, and it can be inferred that transcriptional regulation that relies on epigenetic mechanism may contribute to the effects of heroin addiction. Previous studies had revealed that missense mutation in human GABRD receptor significantly reduced the surface expression of GABA-A receptors, reduced the GABA current and altered the channel gating frequency, which resulted in an impaired inhibitory neurotransmission. Moreover, the abundance or distribution of GABA-A receptors could affect the drug response (Chuang and Reddy, 2018). Reversible DNA cytosine methylation is a signal of chromatin condensation (Jordan et al., 2007). Thus, we speculated that changing level of chromatin condensation of GABRD in coding regions induced by methylation process might affect the GABA-A receptors features such as GABA sensitivity or ligand-gated chloride-ion channels gating frequency.

Recently, converging evidence showing global and site-specific changes in DNA methylation of other genes was also observed in the context of drug addiction (Lax and Szyf, 2018). For instance, a prior study noted that peripheral lymphocyte DNA from heroin addicts of Caucasian origin exhibited hypermethylation at the promoter region of the opioid receptor Mu 1 gene (Nielsen et al., 2012). Besides, long-term parental methamphetamine exposure in mice influenced offspring behavior and hippocampal DNA methylation at 70 hypermethylated and 39 undermethylated loci (Itzhak et al., 2015). In addition, several immediate early genes, such as activity regulated cytoskeletal-associated protein gene, FBJ osteosarcoma oncogene, nuclear receptor subfamily 4 group A member 1 gene, and early growth response 2, were confirmed to be differentially methylated in the frontal cortex and hippocampus of chronic methamphetamine-treatment mice (Cheng et al., 2015). Moreover, methamphetamine induced Tet methylcytosine dioxygenase gene-dependent DNA hypomethylation (Jayanthi et al., 2018). Furthermore, Zhu et al. (2017b) noted that chronic morphine administration induced hypermethylation of the glucocorticoid receptor (GR) promoter and then downregulated the expression of hippocampal GR. Research also suggested that chronic, heavy alcohol consumption led to changes in DNA methylation patterns.

Interestingly, in the present study, we found that pretreatment with MET significantly increased the reinstatement induced by heroin priming, but had no obvious effects on cues-induced reinstatement. Results of a previous study were partially consistent with ours and showed that MET attenuated cocaine-primed reinstatement had no effect on cues-induced cocaine reinstatement (Wright et al., 2015). This could be attributable to the fact that reinstatement induced by cues is more complicated than drug primed reinstatement. For example, Ambroggi et al. (2008) showed that the basolateral amygdala (BLA) and its projections to the NAc interacted to promote the reward-seeking behavioral response and that cues-evoked excitation of NAc neurons depends on BLA input. Therefore, firing activities and response of NAc neurons to MET is not enough to promote cues-induced heroin-seeking behavior. On the contrary, Tian et al. (2012) reported that MET could significantly weaken the rewarding effects elicited by cocaine. Besides, MET was proved to attenuate cocaine-primed reinstatement (Wright et al., 2015) and cause a robust decrease in cocaine conditioned place preference (CPP) (LaPlant et al., 2010; Tian et al., 2012). Importantly, our study illustrated that 5-Aza-dC injection into the NAc significantly inhibited the heroin-seeking behavior induced by heroin priming, which was consistent with the effects of 5-Aza-dC on addictive behaviors with other abused substances. 5-Aza-dC injection into the medial prefrontal cortex (mPFC) significantly reduced alcohol consumption and alcohol preference, prevented excessive alcohol use in rats (Qiao et al., 2017). Furthermore, 5-Aza-dC injection into hippocampus CA1 area restrained acquisition of cocaine in mice but had no impact on cocaine-induced CPP, and injection into prelimbic cortex blocked cocaine-induced CPP but had no effect on acquisition of cocaine (Han et al., 2010). Moreover, 5-Aza-dC injection into the hippocampal CA1 in rats significantly weakened the consolidation and acquisition of morphine-induced CPP in rats (Zhang et al., 2014), disrupted the reconsolidation of morphine-associated withdrawal memory when injection of 5-Aza-dC into the agranular insular and BLA in rats (Liu et al., 2016). However, other studies has shown the different effects of DNA methylation inhibitors, for example, 5-Aza-dC injection into the prelimbic subregion of mPFC significantly potentiated the retrieval of morphine-induced CPP (Zhang et al., 2014), and intracerebroventricular 5-Aza-dC administration facilitated chronic intermittent ethanol-induced ethanol drinking (Qiang et al., 2014). Moreover, 5-Aza-dC injection into the cerebral ventricles of rats enhanced the reinforcing properties of cocaine (Fonteneau et al., 2017). The discrepancy between our results and these studies could be attributed to many factors, such as the the type of drugs, experimental paradigms, dosages, time points of MET and 5-Aza-dC administration, and the specific brain regions tested. As critical nodes within the mesocorticolimbic circuitry, the NAc plays a central role in reinforcement, motivation and drug seeking, the prelimbic subregion of mPFC is involved in extinction memory, the BLA mediates associative learning for both reward and emotions, and the hippocampus is essential for contextual learning memory (Ma et al., 2019). The neurobiological meanings of the brain regions involved in the plasticity underlying the effect of 5-Aza-dC should be noted. Undoubtedly, these findings revealed that 5-Aza-dC might interfere with the reconsolidation of addiction memory through different brain regions, which suggested a potential therapeutic target for drug addiction.

Another important finding in the present study was that decreased GABRD methylation and enhanced GABRD expression in the NAc after reinstatement from heroin self-administration induced by 5-Aza-dC treatment might partially contribute to decreased DNMT1 and DNMT3A expression. Previous studies had indicated that DNMT3A and DNMT3B in the medial prefrontal cortex were upregulated after alcohol exposure and that this upregulation could be reversed by 5-Aza-dc treatment (Qiao et al., 2017). Besides, 5-Aza-dC injected into left ventricle inhibited nicotine-induced an increase in DNMT3A and global DNA methylation (Ke et al., 2017). It should be note that DNMT1 and DNMT3A were more abundant than DNMT3B in adult brain. Furthermore, the ability to form DNMT-DNA adducts in DNMT1 was similar with DNMT3. Thus, it seemed that 5-Aza-dC treatment prefers to decrease DNMT1 and DNMT3A in the NAc when rats exposed to heroin. Further research should be undertaken to fully explore the role of DNMT isoforms in heroin reward and heroin seeking behavior.

Notably, our results revealed that overexpression of GABRD could inhibit reinstatement of heroin-seeking behavior, while GABRD knockdown could promote heroin-seeking behavior. Coincidentally, Peng et al. (2004) revealed that down-regulation of nonsynaptic GABRD receptor levels in both interneurons and principal cells could lead to increased seizure susceptibility in the hippocampal formation in a temporal lobe epilepsy model. Nie et al. (2011) found that knockdown of GABRD receptors in the medial shell region of the NAc affected alcohol intake. Collectively, our data suggested that expression levels of GABRD in the NAc might regulate the responses to drug-seeking behavior; thus, drugs targeting GABRD could emerge as potential therapeutics for drug addiction.

In summary, the present results demonstrated that GABRD might be a potential biomarker and drug target for heroin addiction and relapse. Furthermore, epigenetic regulation through DNA methylation of GABRD may be the main regulatory mechanism underlying heroin addiction and responsible for the neuroadaptations induced by heroin. Besides, it was found that 5-Aza-dC administration might be a potential treatment for heroin relapse.

## Experimental Procedure

### Animals

Adult male Sprague-Dawley rats weighing 250–300 g were purchased from the Experimental Animal Center of Zhejiang Province (Hangzhou, China). Rats were housed in a temperature- and humidity-controlled room with food and water freely available except when specified, and were maintained under a reversed 12-h light/dark cycle (lights on at 19:00 h, off at 07:00 h). Rat was weighed and handled daily for one week prior to surgery. All animal handling procedures were approved by the Laboratory of Behavioral Neuroscience, Animal Care and Use Committee of the Ningbo Institute of Microcirculation and Henbane (Ningbo, China) and conducted strictly in accordance with the National Institutes of Health (NIH) guidelines for the Care and Use of Laboratory Animals (NIH Publications No. 80–23, revised 1978).

### Drugs

Heroin (diacetylmorphine HCl) was derived from the National Institute of Forensic Science (Beijing, China). The dose of heroin used for the self-administration experiment was chosen according to previous reports (Lai et al., 2014; Sun et al., 2015; Zhu et al., 2017a). The methyl donor l-methionine (MET) and the DNMT inhibitor 5′-aza-2′-deoxycytidine (5-Aza-dC) were purchased from Tocris Bioscience (MO, USA). 25 mg MET was dissolved in 1 ml sterile water and 0.2% dimethyl sulfoxide (DMSO, Sigma-Aldrich) and administered at a dose of 225 mg/kg body weight by subcutaneous injection (s.c.). For intra-NAc injections, 1 mg of 5-Aza-dC was dissolved in 1 ml of sterile water and 0.2% DMSO and microinjected in the bilateral NAc of each rat (1 μg/side).

### Plasmids and Transient Transfections

GABRD cDNA was obtained by PCR amplification (forward primer:GGAGGTAGTGGAATGGATCCCGCC ACCATGGACGTTCTGGGCTGG CTGC, reverse primer:TCACCATGGTGGCGGGATCC ATGGTATACGCCGCCCAGTAG, product size: 1,393 bp) and ligated into the AAV vector GV467 after BamH1 restriction enzyme digestion using the ClonExpress TM II One Step Cloning Kit (Vazyme Biotech, Nanjing, China). The reaction was performed in a total volume of 10 µl containing 1 µl of PCR product and 2.5 µl of vector DNA. Subsequently, 10 µl of ligation mix was transformed into 100 µl TOP10 chemically competent cells (Invitrogen, Carlsbad, CA, USA). An appropriate amount of bacterial solution was spread on an Luria–Bertani (LB) agar plate containing 10 μg/ml ampicillin, and subsequently individual colonies were incubated in a shaking incubator at 30°C for 12–16 h. Clones that contained an insertion of the correct size were subsequently transferred to 10 ml of LB liquid medium containing the corresponding antibiotics and incubated overnight at 37°C. The plasmid was extracted using the Endo-Free midi Plasmid Kit (Omega, Norcross, GE, USA) according to the manufacturer's instructions and finally stably transfected into HEK293T cells in 6-well plates. Cellular green fluorescent protein was observed using a Olympus BX51 Upright Fluorescence Microscope (Olympus, Tokyo, Japan) at 24 h after transfection; a fluorescence rate of over 80% indicated appropriate GABRD overexpression. Cells were harvested 48 h later and stored at −80°C until further use.

The recombinant vector CV272 carrying double-stranded DNA oligo containing the interfering sequence of *GABRD* (CTC​GAG​AAG​GTA​TAT​TGC​TGT​TGA​CAG​TGA​GCG​AGA​CGT​GAG​GAA​CGC​CAT​TGT​CTA​GTG​AAG​CCA​CAG​ATG​TAG​ACA​ATG​GCG​TTC​CTC​ACG​TCC​TGC​CTA​CTG​CCT​CGC​AAT​TG, siRNA target sequence: GGA​CGT​GAG​GAA​CGC​CAT​TGT) was transferred to the competent cells of the yeast strain GS115. Monoclonal colonies were identified by PCR, and positive colonies were then sequenced. The plasmid was extracted using the Qiagen Plasmid Plus Maxi Kit (QIAGEN, Hilden, Germany) according to the manufacturer’s suggested protocols.

### Surgery

Rats were catheterized in the right jugular vein as described previously (Lai et al., 2013). Briefly, rats were anesthetized using sodium pentobarbital (50 mg/kg, Serva) administered by intraperitoneal injection (i.p.), and atropine sulfate (0.3 mg/kg, s.c.) was injected before surgery. A silicon catheter (length 3.5 cm, inner diameter 0.5 mm, outer diameter, 0.94 mm) was plugged inthe right external jugular vein, and the other end of the catheter (10 cm, PE20) was passed subcutaneously to the incision of the back, where it exited into a custom-made fluid connector fixed to a jacket. The catheters were irrigated daily with 0.2 ml each of heparinized saline (100 IU) and cefazolin (0.1 g/ml). After 3 days of recovery, the self-administration procedures were initiated.

For stereotaxic injection, rats were anesthetized using sodium pentobarbital (50 mg/kg, i.p.) and installed in a stereotaxic frame (Stoelting, USA). 5-Aza-dC (1 µl per injection at a rate of 0.5 µl/min) was bilaterally injected into the NAc at the following sites according to the rat brain atlas of Paxinos and Watson (1998) (Paxinos and Watson, 1998): anterior–posterior, −1.4 mm from bregma; medial–lateral, ±1.5 mm from midline; dorsal–ventral, −(7.0–7.5) mm from bregma. The needle was maintained in place for an additional 3 min after each injection to ensure the sufficient diffusion of the solution, and then was slowly withdrawn. A Microinjection pump (MD-1001, Bioanalytical System Inc., IN) was used for all injections into the NAc. Finally, the rats were injected intramuscularly with penicillin (15,000 U daily) for at least 3 days to prevent infection.

### Heroin Self-Administration

Firstly, in order to explore the potential changes of DNA methylation in heroin self-administration rats in the NAc, animal behavioral training was initiated after recovery as previously described (Hong et al., 2020). In brief, the rats were trained to self-administer heroin under a fixed ratio paradigm for 4 h daily in operant chambers which equipped with two nose pokes: an active nose-poke hole and an inactive one (ENV-114 M, Med Associates, St. Albans, VT). Each response in the active hole was immediately reinforced with an infusion of heroin (0.5 mg/kg) via the pump over 4 s (Lou et al., 2014), at the same time was accompanied by the noise of the infusion pump and illumination of the stimulus light located above the active nose poke for 20 s. The number of responses during this 20 s period was recorded, but there was no delivery of heroin. Responding on the inactive nose-poke produced no programmed consequences. Daily training sessions were conducted over 14 consecutive days. The criterion of stable heroin self-administration was defined as less than 10% of the variability in the number of active nose poking responses that rats touched on the last 3 days (Lai et al., 2013).

### Yoked–Heroin Self-Administration and Saline Procedure

Both yoked-heroin self-administration (Yoke Heroin) and yoked-saline groups (Control) were tested simultaneously with the heroin self-administration group (Heroin self-administration) in different conditions. The yoked-heroin or yoked-saline rat was paired with the heroin self-administration rat. Yoked-heroin rats acquired intravenous infusions of heroin at the same rate, dose and number as rats in the heroin self-administration group. The yoked-saline group received intravenous infusions of saline at the same rate and number as the heroin self-administration group. Nose-pokes by the yoked rats were recorded but had no scheduled consequences.

### Extinction and Reinstatement of Heroin Self-Administration

After heroin self-administration training sessions, the animals underwent daily 2 h extinction sessions for 12 days. During the extinction phase, the rats were replaced in the operant chambers for 2 h without conditioned cues and heroin, and responses in the active hole were recorded, but there is no programmed consequences regardless of pressing the active or inactive pokes. Rats pressing the active nose-poke for less than 10% of the average active nose-poke responses during maintenance was determined to reach the extinction criterion.

Reinstatement tests were conducted 24 h after the last extinction training. During cues-induced reinstatement, rats were exposed to the same light and tone cues as those used during the period of heroin self-administration; active and inactive responses were recorded, but heroin was not available. During the heroin priming-induced reinstatement test, the rats were first given a single injection of heroin (0.25 mg/kg, s.c.) at 30 min before the reinstatement test session and then were exposed to the conditioned light and tone cues associated with heroin infusion for 5 s. The subsequent active nose-poke response led to the presentation of the conditioned stimulus, but heroin infusion was not available. Nose-pokes during the reinstatement were accumulated over 2 h.

### Locomotor Activity Assessment

After extinction session, acrylic locomotor monitoring cages (AccuScan Instruments, Inc., Columbus, OH) were used to measure the horizontal locomotor activities of rats, each of which contained 16 photocell beams measuring the horizontal distance traveled. After 1 h of habituation to the chamber, beam breaks were continuously recorded once every 10 min for 2 h using the VersaMax/Digiscan System Software (AccuScan Instruments, Inc.).

### Experimental Grouping and Treatments Received

*Experiment 1* During the heroin self-administration training, rats were divided into three groups: i) yoked-saline group, which received saline injections (Control, *n* = 10) for 14 days, ii) heroin self-administration group (*n* = 10), which received heroin self-administration for 14 days, and iii) yoked-heroin group, which received amounts of heroin equal to those received self-administration over the same course of time (Yoke heroin, *n* = 10).

**FIGURE 12:**
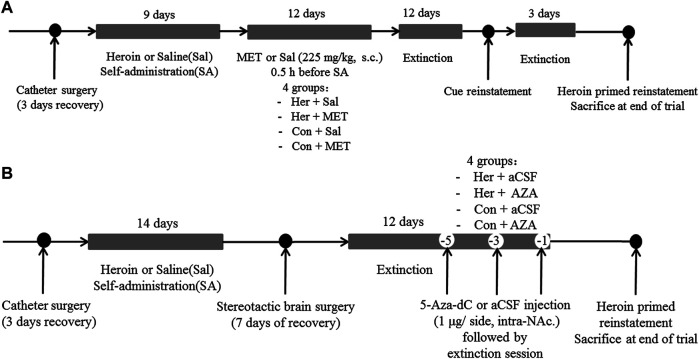
Experimental design. **(A)** Experiment grouping and timeline for MET treatment. Rats were trained for heroin self-administration and subsequently injected with MET (225 mg/kg, s.c.) or saline at 0.5 h prior to heroin self-administration training for 12 consecutive days from the 10th day of training. This was followed by 12 days of extinction training and cues-induced or heroin priming reinstatement.i) heroin self-administration and MET-treated rats (Her + MET, *n* = 5), ii) heroin self-administration and saline-treated rats (Her + Sal, *n* = 5), iii) saline self-administration and MET-treated rats (Con + MET, *n* = 5), and iv) saline self-administration and saline-treated rats (Con + Sal, *n* = 5). **(B)** Experiment grouping and timeline for 5-Aza-dC treatment. Rats underwent bilateral NAc microinjection of 5-Aza-dC or aCSF (1 μl/side, 1 mg/ml, intra-NAc) in the last 1, 3, and 5 days of the extinction training followed by 2 h of heroin priming reinstatement. i) heroin self-administration and 5-Aza-dC treated rats (Her + AZA, *n* = 5), ii) heroin self-administration and aCSF-treated rats (Her + aCSF, *n* = 5), iii) saline self-administration and 5-Aza-dC treated rats (Con + AZA, *n* = 5), and iv) saline self-administration and aCSF-treated rats (Con + aCSF, *n* = 5). MET: l-methionine; s.c.: subcutaneous injection; aCSF: artificial cerebrospinal fluid solution; AZA: 5-Aza-dC, 5-Aza-2-deoxycytidine.

*Experiment 2* For examining the effect of MET on heroin self-administration and cues-induced or heroin priming reinstatement of heroin-seeking, rats were divided into four groups: i) heroin self-administration and MET-treated rats (Her + MET, *n* = 5), ii) heroin self-administration and saline-treated rats (Her + Sal, *n* = 5), iii) saline self-administration and MET-treated rats (Con + MET, *n* = 5), and iv) saline self-administration and saline-treated rats (Con + Sal, *n* = 5) (Figure 12A). Rats were injected with MET (225 mg/kg, s.c.) or saline at 0.5 h prior to heroin self-administration training for 12 consecutive days from the 10th day of training; the dose was chosen based on a previous study (Wright et al., 2015). The self-administration behaviors of rats during this training were recorded. Then rats underwent the extinction training for 12 consecutive days, followed by reinstatement tests to assess cues-induced or heroin priming seeking behavior.

*Experiment 3* To confirm the effect of 5-Aza-dC on DNA methylation and heroin priming reinstatement of heroin-seeking, rats were divided into four groups: i) heroin self-administration and 5-Aza-dC treated rats (Her + AZA, *n* = 5), ii) heroin self-administration and artificial cerebrospinal fluid (aCSF)-treated rats (Her + aCSF, *n* = 5), iii) saline self-administration and 5-Aza-dC treated rats (Con + AZA, *n* = 5), and iv) saline self-administration and aCSF-treated rats (Con + aCSF, *n* = 5) (Figure 12B). Rats underwent bilateral NAc microinjection of 5-Aza-dC or aCSF in the last 1, 3, and 5 days of the extinction training, each followed by a 2 h extinction session. For intra-NAc treatments, 5-Aza-dC (1 μg/side, 1 mg/mL) or aCSF was infused through an injection cannula placed within the guide cannula. The doses of 5-Aza-dC were defined based on a previous study (Qiao et al., 2017).

**FIGURE 13:**
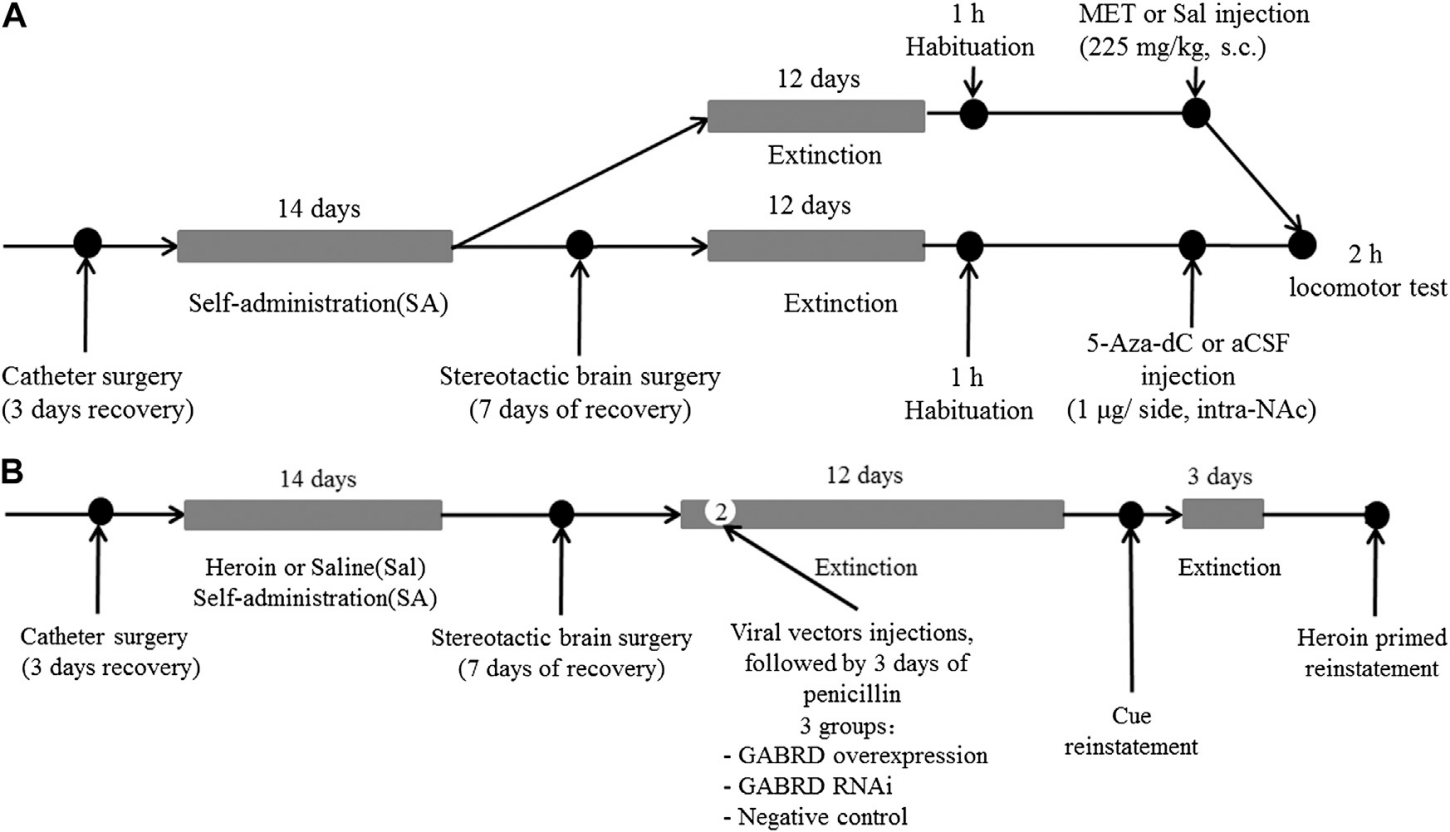
Experimental design **(A)** Timeline for locomotor test (*n* = 7 per group). After extinction training from heroin self-administration, rats were habituated to the AccuScan chamber for 1 h and then injected with MET (225 mg/kg, s.c.), 5-Aza-dC (1 μg/side, intra-NAc), or vehicle (s.c., saline; intra-NAc, aCSF). Horizontal locomotor activities were recorded for 2 h. **(B)** Timeline for microinjection of recombinant AAV vectors targeting GABRD. On the second day of the extinction test, AAV vectors for GABRD overexpression, siRNA, or negative control were microinjected into the NAc of rats (*n* = 8 per group). After 12 days of extinction testing, a 2 h cues-induced or heroin priming reinstatement test was performed. i) overexpression of GABRD (GABRD overexpression), ii) knock-down of GABRD (GABRD RNAi), and iii) negative virus control group (Negative control). MET: l-methionine; s.c.: subcutaneous injection; aCSF: artificial cerebrospinal fluid solution; 5-Aza-dC: 5-Aza-2-deoxycytidine.

*Experiment 4* To investigate the effect of MET or 5-Aza-Dc treatment on locomotor activity, rats that had undergone extinction training from heroin self-administration were habituated to the AccuScan chamber for 1 h and then injected with MET (225 mg/kg, s.c., *n* = 7), 5-Aza-dC (1 μg/side, intra-NAc, *n* = 7), or vehicle (s.c., saline; intra-NAc, aCSF, *n* = 7) and replaced back into the AccuScan chamber (Figure 13A). Horizontal locomotor activities were recorded for 2 h.

*Experiment 5* To investigate whether changes in GABRD expression would affect changes in heroin reinstatement of heroin-seeking behavior, rats were randomly divided into three groups after heroin self-administration training: i) overexpression of GABRD (GABRD overexpression, *n* = 8), ii) knock-down of GABRD (GABRD RNAi, *n* = 8), and iii) negative virus control group (Negative control virus, *n* = 8). On the second day of the extinction test, AAV vectors for GABRD overexpression, RNAi, or negative control (1  μl each) were microinjected into the NAc of rats using a 5  μl syringe needle for bilaterally infusion at a rate of 0.5  μl/min. The needle was maintained in place for an additional 3 min after each injection to ensure the sufficient diffusion of the solution into the brain tissue. After 12 days of extinction testing, a 2 h cues-induced or heroin priming reinstatement test was performed (Figure 13B).

### Tissue Processing

Once all testing was completed, rats were anesthetized using sodium pentobarbital (50 mg/kg, intramuscular) and rapidly decapitated. Brain tissues were dissected from the bregma using a rat brain matrix [2.2–0.8 mm according to coordinates from Paxinos and Watson (1998) rat brain atlas] (1.4 ± 0.1 mm thickness), and serial 1 mm brain sections were cut (Hong et al., 2020). The NAc were harvested bilaterally using a stainless-steel cannula (1.5 mm in diameter). Tissues were stored at −80°C until further use.

### Illumina HiSeq

Genomic DNA was extracted from the NAc using the QIAamp DNA Mini kit (Qiagen, Hilden, Germany). DNA was sheared and then purified using the QIAquick PCR purification kit, and libraries were constructed using the Illumina library preparation kit. Thereafter, the QIAquick PCR purification kit was used to purify the libraries, and the EpiTect bisulfite kit (Qiagen, Hilden, Germany) was suitable for bisulfite conversion. Bisulfite converted libraries were amplified using PfuTurbo HotStart DNA polymerase (Agilent Technologies, CA, USA), size selected (200–300 bp), quantified using qPCR, and run through the Illumina HiSeq 2000 platform as previously described (Dehghanizadeh et al., 2018). The downstream analysis of the obtained sequencing data mainly involved four aspects of the programs/sequencing reads: Fastqc Control, Sequencing Depth Statistics, aligned to the genome, and differentially methylated region (DMR) or site analysis.

### Double Luciferase Reporter Assay

Double luciferase reporter assay was carried out to verify the enhancer activity of the target sequence and examine the effect of methylation on its activity. Reporter gene vectors (pGL3-promoter) carrying GABRD DNA fragments (GABRD: chr5:172809066–172809129) were constructed as follows. The pGL3-promoter was digested with NheI/XhoI, and the recombinant plasmids were transformed into TOP10 competent cells. The correct clones were subjected to sequencing, and the plasmid was extracted using TIANprep Mini Plasmid Kit (Tiangen Biotech, Beijing, China) after verification by sequencing. The constructed plasmids were then methylated using the CpG methyltransferase M.SssI Kit (Thermo, MA, USA) to establish the methylated groups. Thereafter, the EpiJET DNA Methylation Analysis Kit was used to perform the methylation test. HEK293T cells were cultured in Dulbecco's modified Eagle medium (Gibco, Invitrogen, Paisley, United Kingdom) with 10% fetal bovine serum (Gibco, Invitrogen, Paisley, United Kingdom) and 1% penicillin/streptomycin (Gibco, Invitrogen, Paisley, United Kingdom). HEK293T cells were stably co-transfected with recombinant plasmid or methylated plasmids using the Lipofectamine 2000 Transfection Reagent (Invitrogen, Carlsbad, USA) after the cells reached 70% confluence. The reporter assay was carried out using the Dual-Luciferase Reporter Assay system (Promega, Madison City, WI, USA) two days after co-transfection according to the manufacturer's protocol. The recombinant plasmids were divided into 3 groups: i) pGL3-promoter (Pro), ii) pGL3-promoter-GABRD (Pro-GABRD), iii) Me-pGL3-promoter-GABRD (Me-Pro-GABRD).

### Bisulfite Sequencing

GABRD and GABR pi (GABRP) PCR and sequencing primers were designed using the PyroMark Assay Design 2.0 software. The sequences of the forward primers are shown in Table 1. All primers were synthesized by Sangon Biotechnology (Shanghai, China). Genomic DNA (500 ng) was bisulfite treated using the EZ DNA Methylation Gold™ kit (Zymo Research, CA, USA). Bisulfite-converted DNA were amplified as follows: 95°C for 7 min, followed by 40 cycles (95°C for 30 s, 55°C for 30 s, 72°C for 1 min), and 72°C for 5 min. Genes were amplified using Zymo Taq Premix (Zymo Research, CA, USA). The 20 μL PCR reaction was setup as follows: 10 µl Zymo Taq Premix, 6.5 µl DNase/RNase-free water, 1 µl primer pairs (20 pM), and 2.5 μL DNA. The PCR products were run on an Agilent Bioanalyser 2100 to check the specificity of primers according to the Agilent DNA 1000 kit manual (Aglient, CA, USA). A new gene run assay was designed using the PyroMark Q48 software, imported into a U disk, and then loaded into PyroMark Q48 Autoprep. Cleaning and injector priming programs were run to check the status of the instrument. Thereafter, the new run was initiated from the main menu, and reagents were pipetted into the designated injector according to the volumes shown on the instrument touchscreen. Cartridge lids were closed and locked after adding the required reagents. The PCR product (10 μl, about 250 ng template) and 3 μl magnetic beads were loaded into the correct wells of the PyroMark Q48 Disc. The disc was locked correctly into the instrument, a new absorber strip was inserted, and then the run was initiated. Finally, the methylation status of each CpG site was analyzed using the Pyro Q CpG software.

**TABLE 1:** Primers for pyrosequencing and RT-qPCR.

Reaction	Gene	Primer	Sequence (5′–3′)
Pyrosequencing	GABRD	Forward	Biotin-GTGGTTAAGGGTAAGAATGAAGAGA
		Reverse	CCC​ATT​AAA​TCC​ACC​CAA​TTT​TAC​TT
		Sequencing	CCACCCAATTTTACTTT
Pyrosequencing	GABRP	Forward	Biotin-ATTTATGGATGATGGGGTGAG
		Reverse	CCA​AAA​AAC​CAA​ACC​TCT​AAA​ACA​TA
		Sequencing	ACA​CTA​ACA​CAC​AAC​AAT​T
RT-qPCR	GABRD	Forward	AAG​TCT​GCC​TGG​TTC​CAT​GAT​GTG
		Reverse	CTG​TGG​AGG​TGA​TGC​GGA​TGC
RT-qPCR	GABRP	Forward	TTC​AAT​GTG​GAG​GTC​AGC​AGA​AGC
		Reverse	GAG​ATG​CTT​GCG​ATG​TCC​AGA​GTC
RT-qPCR	GAPDH	Forward	GAC​ATG​CCG​CCT​GGA​GAA​AC
		Reverse	AGC​CCA​GGA​TGC​CCT​TTA​GT

### Real-Time RT-qPCR

Total RNA was extracted and reverse-transcripted into single-strand cDNA according to the method described previously (Hong et al., 2020). RT-PCR was used to analyze the mRNA levels of GABRD and GABRP with glyceraldehyde 3-phosphate dehydrogenase (GAPDH) for normalization; the nucleotide sequences of the primers are shown in Table 1. The PCR was performed in a LightCycler ^®^ 480 system (Roche Applied Science), and the 2^−ΔCq^ method was used to analyze the relative changes in mRNA expression levels.

### Western Blot

For protein analysis, NAc samples were homogenized in 300 μl ice-cold lysis buffer (SDS: PMSF = 100: 1) and then centrifuged at 12,000 rpm for 20 min at 4°C. Supernatants were collected, and the total protein concentration was measured using the bicinchoninic acid protein assay reagent kit (Thermo Fisher Scientific, Pittsburgh, PA, USA). The protein solutions were mixed with loading buffer, boiled at 100°C for 5 min, and then separated by 10% SDS-polyacrylamide gel electrophoresis and electroblotted onto a polyvinylidene difluoride membrane (Millipore, Massachusetts, USA). Then the membrane was blocked with 5% skimmed milk for 1 h at 37°C. Subsequently, the membrane was incubated overnight at 4 °C with the appropriate antibodies—anti-GABRD (Sigma, SAB5200052), anti-GAPDH (Proteintech, 60004-1-Ig), anti-DNMT1 (Abclonal, A16729), anti-DNMT3A (Abclonal, A2065), and anti-DNMT3B (Abcam, ab2851). Thereafter, membranes were washed three times with PBST (1,000 mL 1×PBS and 1 mL Tween-20) (10 min each) and incubated with secondary antibody (Goat anti-mouse IgG (H + L) − HRP, 1:10,000, Jackson ImmunoResearch) for 2 h at 37 °C and then washed five times with PBST (5 min each). Protein bands were detected using a chemiluminescence detection system (Bio-Rad, Hercules, CA, USA). Protein expression intensities were expressed as ratios relative to that of GAPDH based on gray-scale analysis.

### Statistical Analyses

Statistical analysis was performed using SPSS 16.0 (SPSS, Inc., Chicago, IL, USA) and GraphPad Prism 5.0 (GraphPad Software, Inc.). All data are expressed as the means ± standard deviations (SDs); *p* < 0.05 was considered indicative of a statistically significant difference. Gene methylation levels were analyzed using Pearson's regression analysis and one-way or two-way analysis of variance (ANOVA) followed by Student–Newman–Keuls post hoc comparisons. One-way and two-way ANOVA was used to analyze mRNA or protein expression, with treatment and heroin self-administration as the independent variables. Effects of MET or 5-Aza-dC on the nose-poke responses and infusions during heroin self-administration and reinstatement were analyzed using two-way repeated-measures ANOVA with treatment group and time as factors. Effect of MET and 5-Aza-dC treatment on locomotion activity, and Effect of GABRD overexpression or RNAi on protein expression level of GABRD were compared using Student *t*-test.

## Data Availability Statement

Our Illumina sequencing data to SRA database had been successfully processed and will be released on 2024-10-01, Accession to cite for these SRA data: PRJNA67367. Our SRA records will be accessible with the following link after the indicated release date: https://www.ncbi.nlm.nih.gov/sra/PRJNA673675.

## Ethics Statement

The animal study was reviewed and approved by Laboratory of Behavioral Neuroscience, Animal Care and Use Committee of the Ningbo Institute of Microcirculation and Henbane (Ningbo, China).

## Author Contributions

HL designed the study; QH, WZ, and HL wrote the manuscript; WX, ZL, JL, and WC performed the experiments; HZ, ML, DZ, ZX, and DF analyzed the data.

## Funding

This work was supported by the National Key R&D Program of China (Grant No. 2017YFC1310400), The Natural Science Foundation of Zhejiang (Grant No. LY18H090008), The Natural Science Foundation of China (Grant No. 81671321), and The Ningbo Science and Technology Project (Grant Nos. 2015C110026, 2017A04, 2019A610293, 2019C50076).

## Conflict of Interest

The authors declare that the research was conducted in the absence of any commercial or financial relationships that could be construed as a potential conflict of interest.
